# Effects of childhood obesity on ocular pulse amplitude and intraocular pressure

**DOI:** 10.5935/0004-2749.20230038

**Published:** 2022-03-08

**Authors:** Semih Bolu, İlke Direkçi, Abdulvahit Aşık

**Affiliations:** 1 Department of Pediatric Endocrinology, Adıyaman University Faculty of Medicine, Adıyaman, Turkey; 2 Department of Eye Diseases, Adıyaman University Faculty of Medicine, Adıyaman, Turkey; 3 Department of Pediatrics, Adıyaman University Faculty of Medicine, Adıyaman, Turkey

**Keywords:** Tonometry, ocular, Intraocular pressure, Obesity, Child, Adolescent, Tanometria ocular, Pressão intraocular, Obesidade, Criança, Adolescente

## Abstract

**Purpose:**

To assess intraocular pressure and ocular pulse amplitude changes in obese children and adolescents using dynamic contour tonometry.

**Methods:**

137 cases, 64 obese and 73 healthy children, who were both age-matched and gender-matched, comprised the study population in this cross-sectional study. Children with body mass index values >95% for sex and age were regarded as obese. Participants underwent detailed ophthalmological examinations, including intraocular pressure measurement using a Pascal dynamic contour tonometer. Relationships between intraocular pressure and ocular pulse amplitude measurements and age, sex, obesity, pubertal status, and insulin resistance were investigated.

**Results:**

Bilateral ocular pulse amplitude was lower while intraocular pressure was higher in the obese group than in the control group (p<0.001). No significant relationship was observed between insulin resistance and intraocular pressure or ocular pulse amplitude (p>0.005). No correlation was determined between systolic and diastolic blood pressure, homeostasis model assessment for insulin resistance, or blood lipid levels and intraocular pressure and ocular pulse amplitude.

**Conclusion:**

Our results show that obesity caused an increase in intraocular pressure and a decrease in ocular pulse amplitude independently of insulin resistance in children and adolescents. Prospective studies involving long-term follow-up of cases are now needed to assess the probable adverse effects of these ocular findings in obese children.

## Abstract (pt)

**Objetivo:**

Avaliar a pressão intraocular e as alterações da amplitude do pulso ocular em crianças e adolescentes obesos, usando tonometria de contorno dinâmico.

**Métodos:**

Um total de 137 casos, sendo 64 crianças obesas e 73 crianças saudáveis, pareadas por idade e sexo, compôs a população estudada neste estudo transversal. Crianças com valores de índice de massa corporal superior ao percentil de 95% para seu sexo e idade foram consideradas obesas. Os participantes foram submetidos a exames oftalmológicos detalhados, incluindo a medição da pressão intraocular com um tonômetro de contorno dinâmico Pascal. As relações entre a pressão intraocular e as medidas da amplitude do pulso ocular com a idade, sexo, obesidade, estado puberal e resistência à insulina foram investigadas.

**Resultados:**

A amplitude do pulso ocular bilateral foi menor no grupo obeso do que no grupo controle saudável (p<0,001), enquanto a pressão intraocular foi maior (p<0,001). Não foi observada nenhuma relação significativa entre a resistência à insulina e a pressão intraocular ou a amplitude de pulso ocular (p>0,005). Não foi determinada nenhuma correlação entre a pressão arterial sistólica e diastólica, a avaliação do modelo de homeostase para resistência à insulina ou os níveis de lipídios sanguíneos e a pressão intraocular e a amplitude de pulso ocular.

**Conclusão:**

Os resultados mostraram que a obesidade causou um aumento da pressão intraocular e uma diminuição da amplitude do pulso ocular em crianças e adolescentes, independentemente da resistência à insulina. São necessários agora estudos prospectivos envolvendo o seguimento de longo prazo dos casos, para avaliar os prováveis efeitos adversos desses achados oculares observados em crianças obesas.

## INTRODUCTION

Childhood obesity is an important public health problem in Turkey, as in the rest of the world^(1)^. The prevalence of obesity in children is also growing due to improper eating habits and an inactive lifestyle. Studies have shown that while, in Europe, one child in four aged 6-9 years was overweight or obese in 2008, that figure had increased significantly by 2010, and approximately one in three children in that age group are now overweight or obese^(2)^. According to Turkish figures for 2009, 14.3% of children in the same age group were overweight and 6.5% were obese^(3)^. Childhood obesity leads to significant health problems, such as type 2 diabetes, hypertension, dyslipidemia, cardiovascular disease, premature mortality (<55 years), asthma, sleep apnea, and psychological and social stress^(4)^. Obesity is known to exacerbate the development of cardiovascular disease through large vessel atherosclerosis in the carotid and femoral artery beds^(5)^. At the same time, obesity also adversely affects microvascular functions. Microvascular changes in the retinal and choroid veins can lead to various eye diseases, including glaucoma, diabetic retinopathy, cataract, and age-related maculopathy^(4)^. Studies have reported that altered blood flow is responsible for the pathologic mechanism of eye diseases, such as glaucoma and diabetic retinopathy, and that blood flow fluctuations are more harmful than stable low perfusion^(6)^. Ocular pulse amplitude (OPA), representing the pulsatile wave front produced by blood passing through the eye, is defined as a difference between systolic and diastolic intraocular pressure (IOP) and is regarded as a potential marker of ocular hemodynamics^(7)^. Changes in the choroid vascular bed caused by obesity can be measured using noninvasive methods. Therefore, the purpose of this study was to evaluate changes in IOP and OPA in obese children and adolescents using dynamic contour tonometry (DCT).

## METHODS

A total of 137 cases (58 females and 79 males) referred to the Ophthalmology Polyclinic by the Pediatric Endocrinology Department between January and April 2016 were evaluated. Obese children with a body mass index (BMI) >95% for sex and age and healthy children in the same age range with normal BMI (<85%) were included in this cross-sectional study. Obese cases were divided into groups based on the presence or absence of insulin resistance and pubertal status. Overweight children (BMI of 85%-95%), children with systemic diseases capable of affecting IOP, such as cardiovascular, renal, or neurological diseases, diabetes, Cushing disease, thyroid disease, infectious and/or inflammatory diseases, sleep apnea, pseudotumor cerebri; those receiving pharmacological therapy; children with orbital or ocular diseases, such as previously known glaucoma, orbital mass, severe myopia (>6D), and corneal diseases; and cases with psychological, metabolic, or genetic disorders were excluded from the study.

All cases included in the study underwent detailed ophthalmological examination, including best corrected visual acuity, biomicroscopy of the anterior segment, fundus examination, Pascal DCT (Pascal dynamic contour tonometer; Swiss Microtechnology AG, Port, Switzerland), and IOP measurement. IOP and OPA values were measured using Pascal DCT. Three consecutive IOP readings were obtained for each eye, and mean values were calculated for analysis. All measurements were performed by the same ophthalmologist. The accuracy of each measurement was assessed using a qualitative score provided by the device, and all IOP readings were only recorded if the Q values were between 1 and 3 (Q= 1 optimum; Q= 2 or 3 acceptable; Q= 4 questionable; and Q= 5 or 6 repetition recommended).

Patients were weighed and measured using a digital scale and a wall-mounted Harpender stadiometer, respectively. BMI was calculated using the formula of weight in kilograms divided by the square of the height in meters (kg/m^2^). Obesity in children was defined as BMI above 95% for age and sex based on reference values for the Turkish pediatric population^(8)^. Pubertal stage was defined based on the Tanner classification^(9)^, and cases were divided into two groups: prepubertal (Tanner stage 1) and pubertal (Tanner stages 2-5). Stage 2 in girls was regarded as thelarche while in boys, cases with testicular volumes ≥4 ml were regarded as pubertal. Testicular volumes were measured using a Prader orchidometer consisting of 12 ellipsoid beads rated between 1 and 25ml (1, 2, 3, 4, 5, 6, 8, 10, 12, 15, 20, and 25ml). Testicular volumes were determined by comparing testes with the different-sized models in the orchidometer.

Blood pressure was measured using a digital automatic blood pressure device (Omron^®^ M2 HEM 7121 E, Omron^®^ Healthcare Co., Japan) following a rest period, and was analyzed three times at 10-minute intervals.

Laboratory findings were recorded from patient data. Blood specimens were collected from all children after 12-hour fasting. Blood lipid and fasting glucose levels were calculated using a Beckman Coulter DXC 800/USA biochemical analyzer. Insulin levels were determined using an ADVIA Centaur XP Immunoassay System (Siemens, Germany) analyzer. The homeostasis model assessment of insulin resistance (HOMA-IR) index was used to determine insulin resistance and was calculated using the formula of HOMA-insulin resistance = insulin (mU/L) x glucose (mmol/L) /22.5. Cut-off values of 2.6 for prepubertal boys and 2.2 for prepubertal females and 5.2 for pubertal females and 3.8 for pubertal males were adopted for detection of insulin resistance^(10)^. Approval for the study was granted by the institutional examination board/ethical committee. The research was also conducted in accordance with the principles of the Declaration of Helsinki. Informed consent was obtained from all parents after the details of the study had been explained.

### Statistical analysis

Descriptive statistics was expressed as mean ± standard deviation and median (IQR) values for continuous variables in a table form. Categorical variables were summarized as numbers and percentages. The normality of the distribution of numerical variables was assessed using the Kolmogorov-Smirnov test. The Independent Samples t-test was used in pairwise comparisons between independent groups when numerical variables were normally distributed, and the Mann Whitney U test when they did not exhibit a normal distribution. The Kruskal Wallis test was used to compare more than two independent groups when numerical variables were not normally distributed. Differences between groups were assessed using the Dwass-Steel-Critchlow-Fligner test. The Pearson chi-square test was applied to compare differences between categorical variables. The Spearman Rho correlation coefficient was employed in the analysis of relationships between IOP and OPA in obese children and demographic characteristics and metabolic and biochemical parameters. Statistical significance was set at a p-value less than 0.05.

## RESULTS

A total of 137 cases, comprising 64 obese patients (22 males and 42 females) and 73 healthy normal controls (36 males and 37 females), were included in the study. The mean ages were 12.2 ± 3.3 years in the patient group and 12.8 ± 3.5 in the control group. There was no significant difference between the groups in age or gender (p>0.05). Bilateral OPA was lower while bilateral IOP was higher in the obese group than in the control group (p<0.001; Table 1). There was no pathology in both groups on the detailed biomicroscopy, optic nerve head examination, and retinal nerve fiber evaluation.

**Table 1:** Comparison of intraocular pressure and ocular pulse amplitude values in obese patients and healthy controls

		Group		95% CI		
		Obese subjects (n=64)	Control (n=73)	Upper	Lower	p-value
IOP	Right	17.2 ± 2.3	14.6 ± 2.2	-2.37	-0.67	<0.001
	Left	16.3 ± 2.1	14.2 ± 2.0	-1.98	-0.49	<0.001
OPA	Right	1.7 ± 0.4	2.0 ± 0.6	-0.54	-0.17	<0.001
	Left	1.4 ± 0.3	1.9 ± 0.6	-0.63	-0.28	<0.001

The Independent Samples t-test was used for the variables with normal distribution while Mann Whitney U test was used for the variables without normal distribution. A p-value of <0.05 was considered statistically significant.

OPA= ocular pulse amplitude; BMI-SDS= body mass index standard de­viation score.

No statistically significant relationship was observed between degree of hepatosteatosis in the obese group and OPA (right eye p=0.218, left eye p=0.418) or IOP (right eye p=0.772, left eye p=0.992). During the comparison of obese cases in terms of presence or absence of insulin resistance, mean OPA in the group with insulin resistance was 1.6 and 1.4 in the left eye compared to 1.6 and 1.4 in the non-insulin resistance group, respectively. In the insulin resistance group, the mean IOP was 17.8 in the right eye and 15.7 in the left eye compared to 17.7 and 16.2 in the non-insulin resistance group, respectively. No significant relationship was observed between insulin resistance and OPA or IOP (p>0.005).

The mean testicular volume of prepubertal obese boys was 2.5 ± 0.5 cc while the mean testicular volume of pubertal obese boys was 17.5 ± 7.5 cc. No significant relationship was observed between the pubertal stage and OPA or IOP in obese patients (p>0.005; Table 2).

**Table 2:** Comparison of ıntraocular pressure and ocular pulse amplitude values in Tanner 1 and Tanner 2-5 stages in obese patients

		Group		95% CI		
		Tanner 1 (n=18)	Tanner (2-5) (n=46)	Lower	Upper	p-value
IOP	Right	14.5 ± 1.9	15.5 ± 2.3	-2.28	0.20	0.099
	Left	14.3 ± 1.8	15.0 ± 2.3	-0.30	0.17	0.583
OPA	Right	1.6 ± 0.2	1.7 ± 0.5	-1.92	0.52	0.258
	Left	1.4 ± 0.2	1.5 ± 0.3	-0.29	0.05	0.179

**Figure 1:**
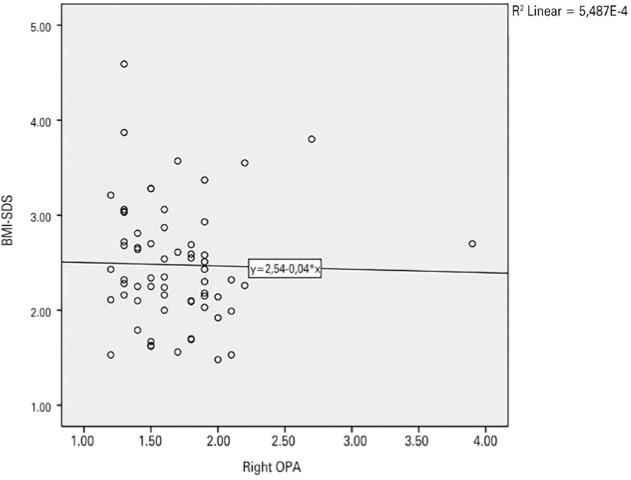
Distribution of the correlation between right OPA and BMI-SDS.

Correlation analysis of OPA and IOP with BMI standard deviation score (BMI-SDS), HOMA-IR, systolic and diastolic blood pressure, and metabolic parameters were performed. Correlation analysis revealed no correlation between OPA or IOP and BMI-SDS, HOMA-IR, systolic and diastolic blood pressure, and blood lipid levels (Table 3). The distribution graphs for the correlation between OPA and BMI-SDS are given in figure 1.

**Table 3:** Correlation analysis of OPA and IOP with HOMA-IR, systolic and diastolic blood pressure, and metabolic parameters

		BMI-SDS	HOMA-IR	SBP	DBP	TG	HDL	LDL	CHOL	FG	FI
**OPA**	Right	-0.158	-0.050	-0.102	0.045	-0.150	-0.214	0.003	-0.174	0.027	-0.074
	Left	-0.005	-0.054	0.002	0.018	-0.091	0.002	-0.141	-0.032	0.024	-0.029
**IOP**	Right	-0.054	-0.164	-0.027	0.079	-0.190	0.165	0.114	0.090	-0.228	-0.116
	Left	0.174	0.095	0.040	-0.054	-0.170	-0.028	0.056	-0.022	-0.010	0.099

CHOL= cholesterol; DBP= diastolic blood pressure; FG= fasting glucose; FI= fasting insulin; HDL= high density lipoprotein, HOMA-IR= homeostasis model assessment for insulin resistance; IOP= intraocular pressure; LDL= low density lipoprotein; OPA= ocular pulse amplitude; SBP= systolic blood pressure; TG= triglycerides.

## DISCUSSION

Obesity is defined as potentially health-damaging abnormal or excessive fat deposition in adipose tissue^(11)^. Obesity causes increased lipid accumulation in adipose tissue, increased release of pro-inflammatory adipocytokines, such as resistin, leptin, interleukin-6, and tumor necrosis factor, and decreased secretion of anti-inflammatory cytokines, such as adiponectin^(12,13)^. Increased pro-inflammatory cytokine production induces the production of free radical species and leads to oxidative stress. Increasing oxidative stress results in inflammation^(14)^.^)^ Additionally, obesity represents the principal risk factor for the development of insulin resistance in children and adolescents^(15)^. Besides an increase in insulin resistance and inflammation in obesity, factors including reduced nitric oxide levels, increased vasoconstrictor molecule levels, such as endothelin 1, angiotensin 2, and arachidonic acid, and decreased microvascular density, leading to tissue hypoxia and ische­mia, have been implicated in microvascular changes to include narrowed arterioles and expanded venular diameter^(16)^. Noninvasive imaging of the retinal vasculature has made it possible to investigate the effects of obesity on microcirculation, and adverse changes in retinal vascular diameter have been linked to metabolic syndrome, risk of diabetes, hypertension, cardiovascular risk factors, coronary heart disease, and stroke^(16)^. Additionally, low OPA values, which indirectly show choroid perfusion and intraocular blood flow, are regarded as a risk factor for severe glaucomatous disorders and visual field flaws^(17)^. The effect of obesity on OPA has been investigated in a few adult studies, but, to the best of our knowledge, the present study is the first one to evaluate the relationship between OPA and childhood obesity. Studies have observed decreased OPA values in adults with high BMI and have reported that these low OPA values may indicate low choroid perfusion and low ocular blood flow^(18,19)^. Consistent with adult studies, the present research determined a significant decrease in OPA values in obese children and adolescents. The low OPA values determined may indicate low choroid perfusion in obese children, similarly to obese adults. OPA values in obese cases were significantly lower than in the control group, even after regulation of insulin resistance. This study finding shows that obesity in children affects OPA independently of insulin resistance.

The relationship between childhood obesity and IOP has been assessed in various studies, but inconsistent results have emerged. Some studies have reported a positive relationship between BMI and IOP^(20,21)^, while others have observed no such relationship^(22,23)^. This study observed statistically higher IOP in obese children and adolescents than in the control group, with IOP values in the obese group still within the normal range for this parameter in children. Biomicroscopy and optic nerve head and retinal nerve fiber layer evaluation did not reveal any features or risk factors for the development of glaucoma in these obese patients compared to the control group. Possibly, this is because patients are not questioned how long they have been obese, and glaucoma is a slow-progressing disease that results from its natural process.

In their study of 49 female and 23 male obese children, Akinci et al.^(20)^ described obesity as an independent risk factor for IOP, and that IOP was higher compared to the healthy control group even after blood pressure regulation. Baran et al.^(21)^ also observed higher IOP in obese children compared to a healthy group, although the difference was very small. Affected IOP in obesity has been attributed to an increase in periorbital fat tissue that prevents choroid perfusion and obstructs ocular blood flow and increased blood viscosity increa­sing outflow in the episcleral veins. Studies have also shown a relationship between IOP and insulin resistance outside obesity. Oh SW et al.^(24)^ reported no significant relationship between IOP and obesity in models in which insulin resistance was excluded, and that insulin resistance may contribute to a positive correlation between IOP and obesity. The present study also obser­ved significantly higher IOP in obese children and adolescents compared to the control group even after insulin resistance regulation. This finding supports the idea that obesity may be a risk factor for IOP independently of insulin resistance.

IOP measurement occupies an important place in the ocular examination. However, IOP measurement is difficult in very young children due to noncompliance. Noncontact tonometry devices overestimate IOP in noncompliant children and thus, are unreliable. One such device, the Goldman applanation tonometer, is regarded as the gold standard in IOP measurement^(25)^. However, it can result in errors in the corneal thickness measurement. The nonapplanation contact tonometer method, Pascal DCT, was used in the present study since it is less dependent on corneal thickness, permits OPA measurement in addition to IOP, and exhibits less inter-and intra-observer variation than the Goldman tonometer^(26)^.

In conclusion, our research shows that obesity in children and adolescents causes an insulin resistance-independent increase in IOP and a decrease in OPA. Further prospective studies involving long-term case follow-ups are necessary to evaluate the potential adverse effects of these ocular findings in obese children and adolescents. Balanced nutrition and regular physical activity from an early age may help protect children from obesity-related ocular diseases.
